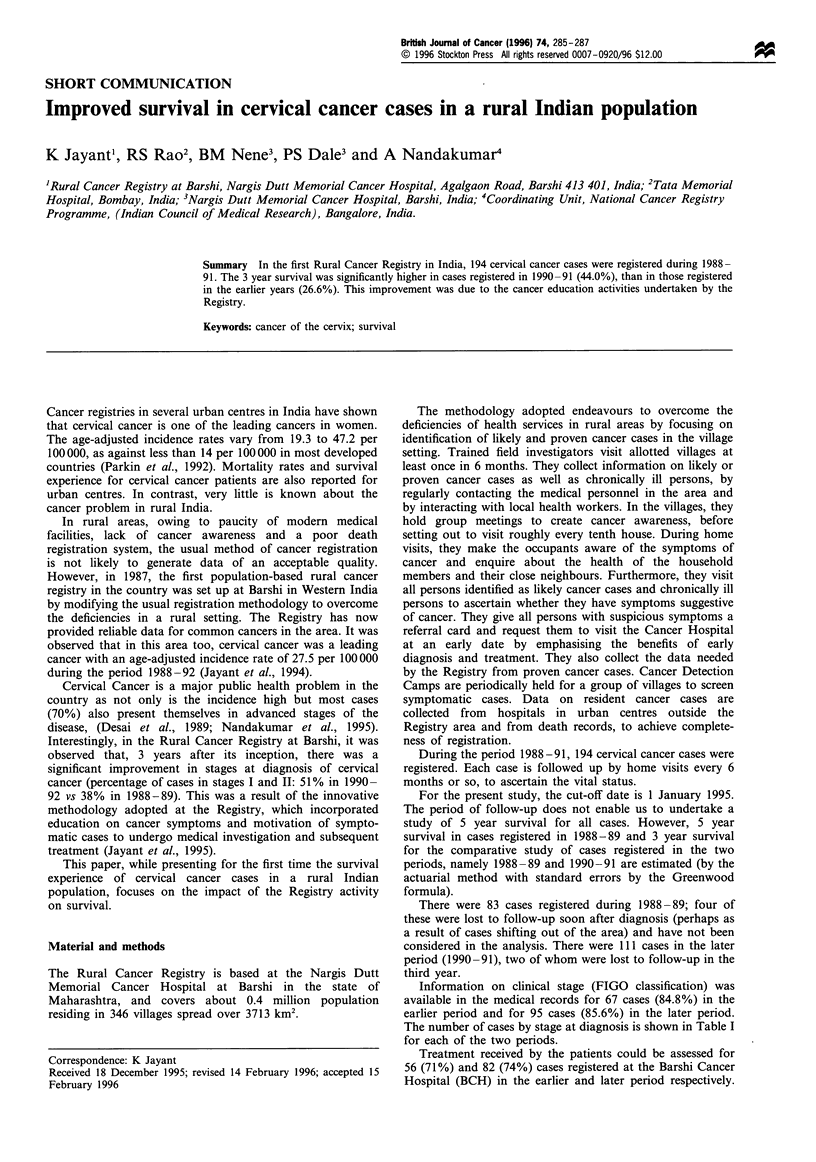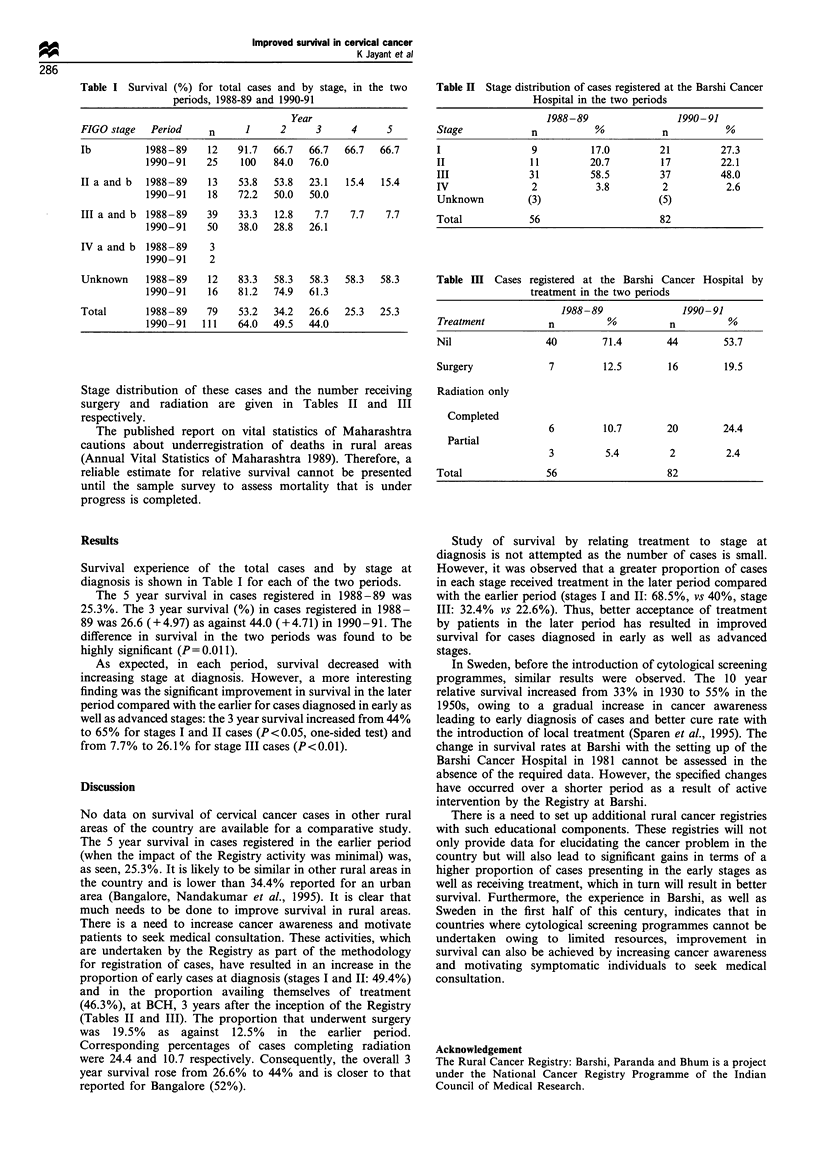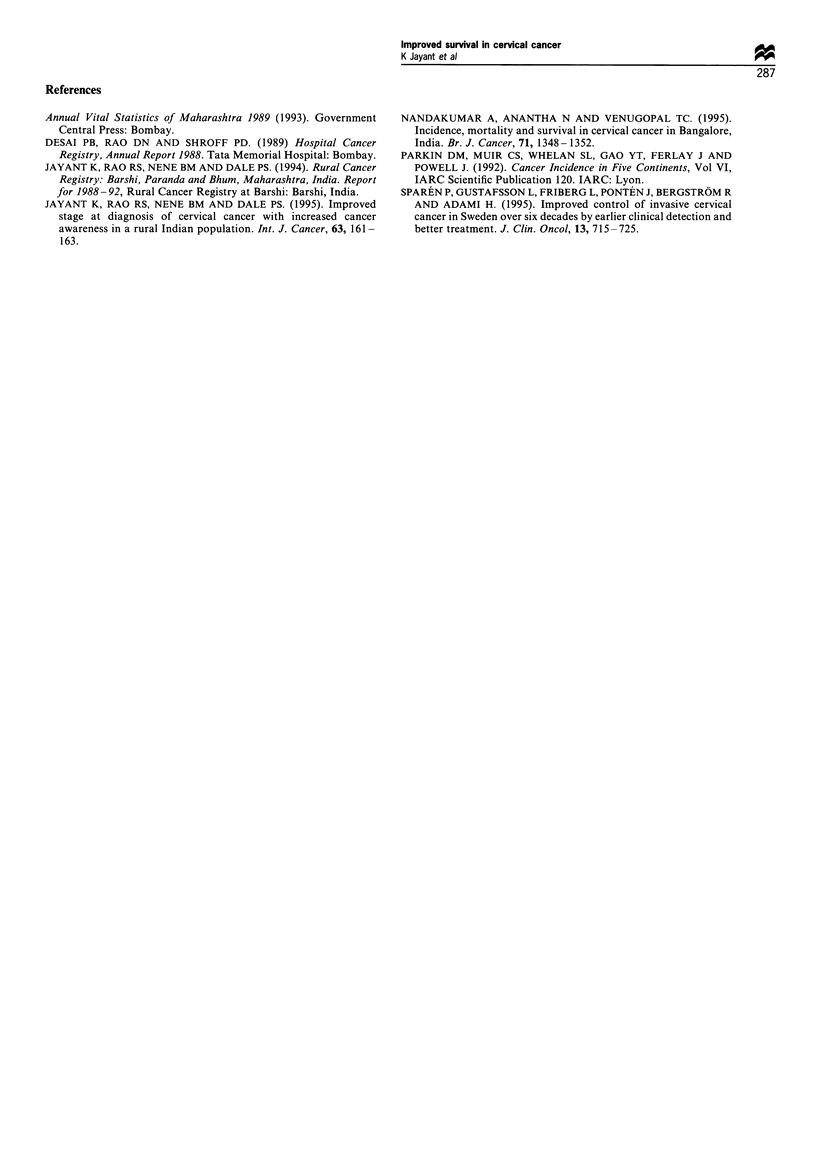# Improved survival in cervical cancer cases in a rural Indian population.

**DOI:** 10.1038/bjc.1996.353

**Published:** 1996-07

**Authors:** K. Jayant, R. S. Rao, B. M. Nene, P. S. Dale, A. Nandakumar

**Affiliations:** Rural Cancer Registry at Barshi, Nargis Dutt Memorial Cancer Hospital, India.

## Abstract

In the first Rural Cancer Registry in India, 194 cervical cancer cases were registered during 1988-91. The 3 year survival was significantly higher in cases registered in 1990-91 (40.0%), than in those registered in the earlier years (26.6%). This improvement was due to the cancer education activities undertaken by the Registry.


British Journal of Cancer (1996) 74, 285-287
© 1996 Stockton Press All rights reserved 0007-0920/96 $12.00

SHORT COMMUNICATION

### Improved survival in cervical cancer cases in a rural Indian population

K Jayant[1], RS Rao[2], BM Nene[3], PS Dale[3] and A Nandakumar[4]

[1]*Rural Cancer Registry at Barshi, Nargis Dutt Memorial Cancer Hospital, Agalgaon Road, Barshi 413 401, India;* [2]*Tata Memorial Hospital, Bombay, India;* [3]*Nargis Dutt Memorial Cancer Hospital, Barshi, India;* [4]*Coordinating Unit, National Cancer Registry Programme, (Indian Council of Medical Research), Bangalore, India.*

**Summary** In the first Rural Cancer Registry in India, 194 cervical cancer cases were registered during 1988-91. The 3 year survival was significantly higher in cases registered in 1990-91 (44.0%), than in those registered in the earlier years (26.6%). This improvement was due to the cancer education activities undertaken by the Registry.

**Keywords:** cancer of the cervix; survival

Cancer registries in several urban centres in India have shown that cervical cancer is one of the leading cancers in women. The age-adjusted incidence rates vary from 19.3 to 47.2 per 100 000, as against less than 14 per 100 000 in most developed countries (Parkin *et al.*, 1992). Mortality rates and survival experience for cervical cancer patients are also reported for urban centres. In contrast, very little is known about the cancer problem in rural India.

In rural areas, owing to paucity of modern medical facilities, lack of cancer awareness and a poor death registration system, the usual method of cancer registration is not likely to generate data of an acceptable quality. However, in 1987, the first population-based rural cancer registry in the country was set up at Barshi in Western India by modifying the usual registration methodology to overcome the deficiencies in a rural setting. The Registry has now provided reliable data for common cancers in the area. It was observed that in this area too, cervical cancer was a leading cancer with an age-adjusted incidence rate of 27.5 per 100 000 during the period 1988-92 (Jayant *et al.*, 1994).

Cervical Cancer is a major public health problem in the country as not only is the incidence high but most cases (70%) also present themselves in advanced stages of the disease, (Desai *et al.*, 1989; Nandakumar *et al.*, 1995). Interestingly, in the Rural Cancer Registry at Barshi, it was observed that, 3 years after its inception, there was a significant improvement in stages at diagnosis of cervical cancer (percentage of cases in stages I and II: 51% in 1990-92 *vs* 38% in 1988-89). This was a result of the innovative methodology adopted at the Registry, which incorporated education on cancer symptoms and motivation of symptomatic cases to undergo medical investigation and subsequent treatment (Jayant *et al.*, 1995).

This paper, while presenting for the first time the survival experience of cervical cancer cases in a rural Indian population, focuses on the impact of the Registry activity on survival.

### Material and methods

The Rural Cancer Registry is based at the Nargis Dutt Memorial Cancer Hospital at Barshi in the state of Maharashtra, and covers about 0.4 million population residing in 346 villages spread over 3713 km$^2$.

Correspondence: K Jayant
Received 18 December 1995; revised 14 February 1996; accepted 15 February 1996

The methodology adopted endeavours to overcome the deficiencies of health services in rural areas by focusing on identification of likely and proven cancer cases in the village setting. Trained field investigators visit allotted villages at least once in 6 months. They collect information on likely or proven cancer cases as well as chronically ill persons, by regularly contacting the medical personnel in the area and by interacting with local health workers. In the villages, they hold group meetings to create cancer awareness, before setting out to visit roughly every tenth house. During home visits, they make the occupants aware of the symptoms of cancer and enquire about the health of the household members and their close neighbours. Furthermore, they visit all persons identified as likely cancer cases and chronically ill persons to ascertain whether they have symptoms suggestive of cancer. They give all persons with suspicious symptoms a referral card and request them to visit the Cancer Hospital at an early date by emphasising the benefits of early diagnosis and treatment. They also collect the data needed by the Registry from proven cancer cases. Cancer Detection Camps are periodically held for a group of villages to screen symptomatic cases. Data on resident cancer cases are collected from hospitals in urban centres outside the Registry area and from death records, to achieve completeness of registration.

During the period 1988-91, 194 cervical cancer cases were registered. Each case is followed up by home visits every 6 months or so, to ascertain the vital status.

For the present study, the cut-off date is 1 January 1995. The period of follow-up does not enable us to undertake a study of 5 year survival for all cases. However, 5 year survival in cases registered in 1988-89 and 3 year survival for the comparative study of cases registered in the two periods, namely 1988-89 and 1990-91 are estimated (by the actuarial method with standard errors by the Greenwood formula).

There were 83 cases registered during 1988-89; four of these were lost to follow-up soon after diagnosis (perhaps as a result of cases shifting out of the area) and have not been considered in the analysis. There were 111 cases in the later period (1990-91), two of whom were lost to follow-up in the third year.

Information on clinical stage (FIGO classification) was available in the medical records for 67 cases (84.8%) in the earlier period and for 95 cases (85.6%) in the later period. The number of cases by stage at diagnosis is shown in Table I for each of the two periods.

Treatment received by the patients could be assessed for 56 (71%) and 82 (74%) cases registered at the Barshi Cancer Hospital (BCH) in the earlier and later period respectively.

**Table I** Survival (%) for total cases and by stage, in the two periods, 1988-89 and 1990-91

| FIGO stage | Period | n | Year 1 | 2 | 3 | 4 | 5 |
|---|---|---|---|---|---|---|---|
| Ib | 1988–89 | 12 | 91.7 | 66.7 | 66.7 | 66.7 | 66.7 |
| | 1990–91 | 25 | 100 | 84.0 | 76.0 | | |
| II a and b | 1988–89 | 13 | 53.8 | 53.8 | 23.1 | 15.4 | 15.4 |
| | 1990–91 | 18 | 72.2 | 50.0 | 50.0 | | |
| III a and b | 1988–89 | 39 | 33.3 | 12.8 | 7.7 | 7.7 | 7.7 |
| | 1990–91 | 50 | 38.0 | 28.8 | 26.1 | | |
| IV a and b | 1988–89 | 3 | | | | | |
| | 1990–91 | 2 | | | | | |
| Unknown | 1988–89 | 12 | 83.3 | 58.3 | 58.3 | 58.3 | 58.3 |
| | 1990–91 | 16 | 81.2 | 74.9 | 61.3 | | |
| Total | 1988–89 | 79 | 53.2 | 34.2 | 26.6 | 25.3 | 25.3 |
| | 1990–91 | 111 | 64.0 | 49.5 | 44.0 | | |

Stage distribution of these cases and the number receiving surgery and radiation are given in Tables II and III respectively.

The published report on vital statistics of Maharashtra cautions about underregistration of deaths in rural areas (Annual Vital Statistics of Maharashtra 1989). Therefore, a reliable estimate for relative survival cannot be presented until the sample survey to assess mortality that is under progress is completed.

**Table II** Stage distribution of cases registered at the Barshi Cancer Hospital in the two periods

| Stage | 1988–89 n | % | 1990–91 n | % |
|---|---|---|---|---|
| I | 9 | 17.0 | 21 | 27.3 |
| II | 11 | 20.7 | 17 | 22.1 |
| III | 31 | 58.5 | 37 | 48.0 |
| IV | 2 | 3.8 | 2 | 2.6 |
| Unknown | (3) | | (5) | |
| Total | 56 | | 82 | |

**Table III** Cases registered at the Barshi Cancer Hospital by treatment in the two periods

| Treatment | 1988–89 n | % | 1990–91 n | % |
|---|---|---|---|---|
| Nil | 40 | 71.4 | 44 | 53.7 |
| Surgery | 7 | 12.5 | 16 | 19.5 |
| Radiation only | | | | |
| Completed | 6 | 10.7 | 20 | 24.4 |
| Partial | 3 | 5.4 | 2 | 2.4 |
| Total | 56 | | 82 | |

### Results

Survival experience of the total cases and by stage at diagnosis is shown in Table I for each of the two periods.

The 5 year survival in cases registered in 1988–89 was 25.3%. The 3 year survival (%) in cases registered in 1988–89 was 26.6 (+4.97) as against 44.0 (+4.71) in 1990–91. The difference in survival in the two periods was found to be highly significant ($P=0.011$).

As expected, in each period, survival decreased with increasing stage at diagnosis. However, a more interesting finding was the significant improvement in survival in the later period compared with the earlier for cases diagnosed in early as well as advanced stages: the 3 year survival increased from 44% to 65% for stages I and II cases ($P<0.05$, one-sided test) and from 7.7% to 26.1% for stage III cases ($P<0.01$).

### Discussion

No data on survival of cervical cancer cases in other rural areas of the country are available for a comparative study. The 5 year survival in cases registered in the earlier period (when the impact of the Registry activity was minimal) was, as seen, 25.3%. It is likely to be similar in other rural areas in the country and is lower than 34.4% reported for an urban area (Bangalore, Nandakumar *et al.*, 1995). It is clear that much needs to be done to improve survival in rural areas. There is a need to increase cancer awareness and motivate patients to seek medical consultation. These activities, which are undertaken by the Registry as part of the methodology for registration of cases, have resulted in an increase in the proportion of early cases at diagnosis (stages I and II: 49.4%) and in the proportion availing themselves of treatment (46.3%), at BCH, 3 years after the inception of the Registry (Tables II and III). The proportion that underwent surgery was 19.5% as against 12.5% in the earlier period. Corresponding percentages of cases completing radiation were 24.4 and 10.7 respectively. Consequently, the overall 3 year survival rose from 26.6% to 44% and is closer to that reported for Bangalore (52%).

Study of survival by relating treatment to stage at diagnosis is not attempted as the number of cases is small. However, it was observed that a greater proportion of cases in each stage received treatment in the later period compared with the earlier period (stages I and II: 68.5%, *vs* 40%, stage III: 32.4% *vs* 22.6%). Thus, better acceptance of treatment by patients in the later period has resulted in improved survival for cases diagnosed in early as well as advanced stages.

In Sweden, before the introduction of cytological screening programmes, similar results were observed. The 10 year relative survival increased from 33% in 1930 to 55% in the 1950s, owing to a gradual increase in cancer awareness leading to early diagnosis of cases and better cure rate with the introduction of local treatment (Sparen *et al.*, 1995). The change in survival rates at Barshi with the setting up of the Barshi Cancer Hospital in 1981 cannot be assessed in the absence of the required data. However, the specified changes have occurred over a shorter period as a result of active intervention by the Registry at Barshi.

There is a need to set up additional rural cancer registries with such educational components. These registries will not only provide data for elucidating the cancer problem in the country but will also lead to significant gains in terms of a higher proportion of cases presenting in the early stages as well as receiving treatment, which in turn will result in better survival. Furthermore, the experience in Barshi, as well as Sweden in the first half of this century, indicates that in countries where cytological screening programmes cannot be undertaken owing to limited resources, improvement in survival can also be achieved by increasing cancer awareness and motivating symptomatic individuals to seek medical consultation.

**Acknowledgement**
The Rural Cancer Registry: Barshi, Paranda and Bhum is a project under the National Cancer Registry Programme of the Indian Council of Medical Research.

### References

*Annual Vital Statistics of Maharashtra 1989* (1993). Government Central Press: Bombay.

DESAI PB, RAO DN AND SHROFF PD. (1989) *Hospital Cancer Registry, Annual Report 1988*. Tata Memorial Hospital: Bombay.

JAYANT K, RAO RS, NENE BM AND DALE PS. (1994). *Rural Cancer Registry: Barshi, Paranda and Bhum, Maharashtra, India. Report for 1988–92*, Rural Cancer Registry at Barshi: Barshi, India.

JAYANT K, RAO RS, NENE BM AND DALE PS. (1995). Improved stage at diagnosis of cervical cancer with increased cancer awareness in a rural Indian population. *Int. J. Cancer*, **63**, 161–163.

NANDAKUMAR A, ANANTHA N AND VENUGOPAL TC. (1995). Incidence, mortality and survival in cervical cancer in Bangalore, India. *Br. J. Cancer*, **71**, 1348–1352.

PARKIN DM, MUIR CS, WHELAN SL, GAO YT, FERLAY J AND POWELL J. (1992). *Cancer Incidence in Five Continents*, Vol VI, IARC Scientific Publication 120. IARC: Lyon.

SPARÉN P, GUSTAFSSON L, FRIBERG L, PONTÉN J, BERGSTRÖM R AND ADAMI H. (1995). Improved control of invasive cervical cancer in Sweden over six decades by earlier clinical detection and better treatment. *J. Clin. Oncol*, **13**, 715–725.